# Novel Self-shrinking Mask for Sub-3 nm Pattern Fabrication

**DOI:** 10.1038/srep29625

**Published:** 2016-07-12

**Authors:** Po-Shuan Yang, Po-Hsien Cheng, C. Robert Kao, Miin-Jang Chen

**Affiliations:** 1 Department of Materials Science and Engineering, National Taiwan University, 1, Roosevelt Road, Sec. 4, Taipei, 106, ROC Taiwan

## Abstract

It is very difficult to realize sub-3 nm patterns using conventional lithography for next-generation high-performance nanosensing, photonic, and computing devices. Here we propose a completely original and novel concept, termed self-shrinking dielectric mask (SDM), to fabricate sub-3 nm patterns. Instead of focusing the electron and ion beams or light to an extreme scale, the SDM method relies on a hard dielectric mask which shrinks the critical dimension of nanopatterns during the ion irradiation. Based on the SDM method, a linewidth as low as 2.1 nm was achieved along with a high aspect ratio in the sub-10 nm scale. In addition, numerous patterns with assorted shapes can be fabricated simultaneously using the SDM technique, exhibiting a much higher throughput than conventional ion beam lithography. Therefore, the SDM method can be widely applied in the fields which need extreme nanoscale fabrication.

Because of the rapid evolution of nanosensors, nanophotonics, and semiconductor technology down to the sub-10 nm technology node, the research of sub-10 nm patterning has attracted much attention in recent years^1^,^2^,^3^,^4^,^5^,^6^. For instance, sub-10 nm electromagnetic hot spots with extreme near-field confinement are crucial to plasmonic applications^6^,^7^,^8^. Nanoscaled graphene, quantum point contacts, and quantum dot devices for high level computing require precise sub-10 nm patterning^9^. Thus a variety of approaches including the helium and neon ion beam lithography^10^,^11^, electron beam lithography^12^,^13^,^14^,^15^,^16^, advanced scanning probe lithography^17^, nanoimprint lithography^18^,^19^, and extreme ultraviolet lithography (EUV)^20^,^21^,^22^, have been proposed in order to resolve the difficulties of the sub-10 nm patterning. Nonetheless, these methods all have their individual problems which are difficult to be overcome. For example, ion beam, electron beam, and scanning probe lithography are capable of high-resolution patterning, but their throughput is typically low^23^,^24^,^25^,^26^,^27^. Although the high-voltage electron-beam lithography based on an aberration-corrected scanning transmission electron microscope (TEM) is a very promising approach to achieve the patterns down to 2 nm^15^,^16^, large-area patterning is not feasible currently using this method because a large sample cannot be put into the TEM holder. Nanoimprint lithography suffers from the surface sticking issue and poor mold condition^19^. As for the EUV lithography, although a short wavelength of 13.5 nm provides a high potential for nanopatterning, EUV lithography has not yet to be proven effective and needs further development due to the challenges from photoresist, mask, optical reflectivity, and source power^21^,^28^,^29^. Consequently, quadruple patterning is under high consideration for the 7 nm technology node in semiconductor industry due to the delay of the EUV lithography. However, how far the quadruple patterning can go depends on the cost, overlay control, and the cost-effectiveness of the extreme scaling^30^. Another technique such as ALD (atomic layer deposition) spacer technique has also been proposed to reduce pitch size^31^. However, the spacer is prone to collapse if the spacer thickness is less than 5 nm^31^. The shadow (angle) evaporation is also capable of obtaining sub-10 nm gaps based on the poor conformality of evaporation^32^. Nevertheless, it is not easy to precisely control the patterning by this approach.

It is difficult to achieve the patterns with a linewidth down to sub-3 nm scale using the conventional lithography based on focusing optical, electron, or ion beams. Hence, we conceive that instead of using focused beam approaches, shrinking the pattern itself will be a more practical and facile way to achieve the sub-3 nm fabrication. In this study, we demonstrate a new concept and method, entitled self-shrinking dielectric mask (SDM), to realize sub-3 nm patterns. The SDM method starts from an initial pattern on a hard dielectric mask, which could be defined by conventional optical, ion beam, or electron beam lithography. Afterwards the sample is exposed to the ion irradiation for a couple of minutes, and then the linewidth of the initial patterns on the mask will shrink during the ion irradiation. Together with a high aspect ratio, a critical dimension down to 2.1 nm has been realized with the SDM method in this study. Besides, a large area of nanopatterns can be fabricated simultaneously by the ion irradiation on SDM, instead of using sequential line-by-line writing such as those in the focused ion beam and electron beam lithography. Thus, a considerable amount of process time can be saved and the throughput can be greatly improved. Actually, one of the practical applications of SDM is shown in the supporting information. We have implemented the SDM method to define a sub-10 nm gate in the junctionless Si transistors on silicon-on-insulator substrate. Therefore, the SDM technique is an effective, high-throughput, and practically applicable nanofabrication technique to tailor the structures with the feature sizes down to a few nanometers, which can be applied not only in the high-performance computing devices, but also in the advanced nanosensors and nanoplasmonics. Accordingly, SDM shows a strong and promising potential to have a role in the next-generation high-throughput sub-3 nm fabrication.

## Results

**Figure 1:**
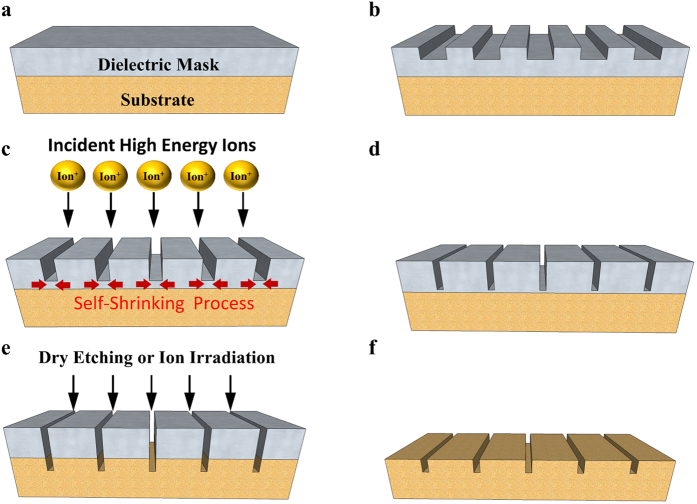
The SDM method. (**a**) The substrate is deposited with a hard dielectric mask such as Al_2_O_3_. (**b**) An initial pattern (e.g. a line array) is defined by the conventional lithography such as optical, ion and electron beam lithography, etc. (**c**) Scheme illustrates the self-shrinking process on the dielectric mask during the ion irradiation. (**d**) A hard mask with the target linewidth is obtained when the ion irradiation is stopped. (**e**) Then the patterns are transferred to the substrate by subsequent dry etching such as reactive ion etching or further ion exposure. (**f**) The dielectric mask is etched away and then the nanopatterns on the substrate are obtained.

Figure 1 depicts the SDM method schematically. First, a hard dielectric layer such as Al_2_O_3_ is deposited on the substrate (Fig. 1a), and then an initial pattern (e.g. a line array) is defined by conventional lithography (Fig. 1b). Afterwards, the sample is exposed to the ion irradiation for a couple of minutes, and the gap will shrink during the ion irradiation (Fig. 1c). The ion irradiation will be stopped once the target linewidth of nanogap is reached, and then a hard mask with the target linewidth is obtained (Fig. 1d). With this hard dielectric mask, we can transfer the patterns to the substrate by subsequent reactive ion etching or by further ion irradiation (Fig. 1e). Finally, the hard mask is removed away by etching after the patterns are transferred to the substrate (Fig. 1f).

**Figure 2:**
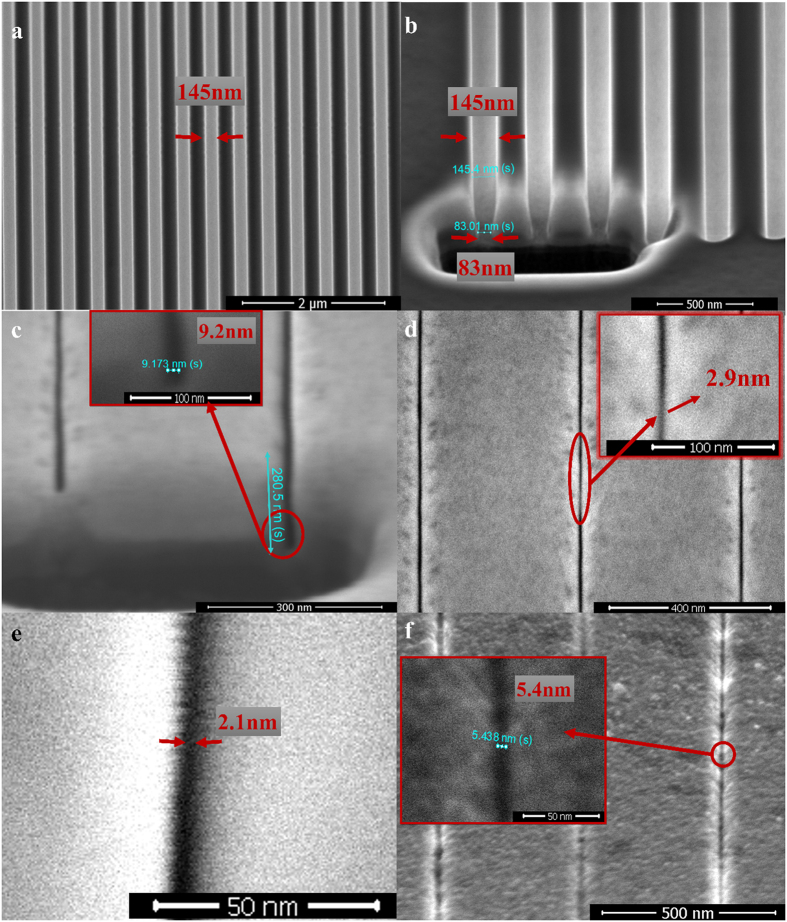
SEM images of the nanopatterns fabricated by the SDM method. (**a**) An initial pattern (an Al_2_O_3_ line array) fabricated by the focused Ga ion beam. (**b**) Cross section of the initial pattern with a V-shaped structure before the ion irradiation. (**c**) Cross section of the shrunk line array with a high aspect ratio of ~30. The nanogap on the left side of the figure was filled by the debris of Al_2_O_3_ during the fabrication of the cross section using the focused Ga ion beam. A protection layer was not deposited upon the dielectric mask for the fabrication of the cross section because it would fill the gap so that we could not observe the gap width clearly. In fact, the absence of the protection layer does not result in the change of the lateral width of the nanogaps. It may only influence the surface morphology due to the sputtering away of the surface. (**d**) The line array shrunk after the ion irradiation, and a linewidth as low as 2.9 nm was shown in the inset. (**e**) A top view of the nanogap in the line array after the further ion exposure, exhibiting a minimum linewidth as low as 2.1 nm. (**f**) Line patterns were transferred to the substrate by subsequent ion irradiation, revealing a critical dimension less than 10 nm (minimum linewidth is ~5.4 nm). It should be noticed that the patterns were transferred from the hard mask shown in Fig. 3f. It is seen that the linewidth of patterns transferred to the substrate (f) is comparable to that of the hard mask (Fig. 3f).

The scanning electron microscope (SEM) pictures of nanopatterns fabricated by SDM method are shown in Fig. 2. In Fig. 2a, an Al_2_O_3_ line array with ~145 nm gap width was fabricated by focused Ga ion beam to act as the initial pattern for SDM. Figure 2b shows the cross section of the line array with a V**-**shaped geometry (a top width of ~145 nm and a bottom width of ~83 nm) which is mostly due to the redeposition effect^33^. This is hard to avoid when using focused Ga ion beam to fabricate deep nanopatterns. Afterwards, the Al_2_O_3_ line array was exposed to the Ga ions, and the gap size started to shrink during the ion irradiation. After a couple of minutes, the gap shrunk to the sub-10 nm scale. Figure 2c shows an example of the cross section of the nanogap after the ion irradiation. The gap was estimated to be ~280 nm in depth with an opening of only 9.2 nm in width. This aspect ratio is ~30 which is quite extraordinary for sub-10 nm patterns^34^. Figure 2d illustrates a tiny gap size on the Al_2_O_3_ mask of a critical dimension as low as 2.9 nm after the subsequent ion exposure. Further ion irradiation leads to a minimum gap width down to 2.1 nm as shown in Fig. 2e. This narrow linewidth is much smaller than the spot size of the focused Ga ion beam even with the lowest beam current (The smallest spot size is ~7 nm at the lowest beam current of ~1.1 pA for FEI Helios Nanolab 600i focused ion beam system). In fact, there is no need to focus the ion beam to get nanopatterrns using the SDM technique. The beam current we used in the experiment is as large as 2.5 nA, and the focused spot size at this beam current is about 133 nm^35^. Accordingly, with a 2.5 nA beam current, the optimal resolution for Ga ion beam lithography is not smaller than 133 nm. Nevertheless, a pattern down to 2.1 nm has been achieved by the SDM method, which clearly indicates that focusing the beam is not necessary to get a sub-3 nm pattern using SDM. Additionally, it is known that the ion milling rate is proportional to the beam current^36^. As a result, by using a more than two thousand times larger beam current (2.5 nA beam current rather than 1.1 pA), we have saved over 99.9% of time to fabricate sub-10 nm patterns. Finally, after the patterns were transferred to the substrate underneath the Al_2_O_3_ layer, the Al_2_O_3_ mask was removed by wet etching. An example of the sub-10 nm linewidth on the substrate is shown in Fig. 2f, demonstrating a linewidth as low as ~5.4 nm.

**Figure 3:**
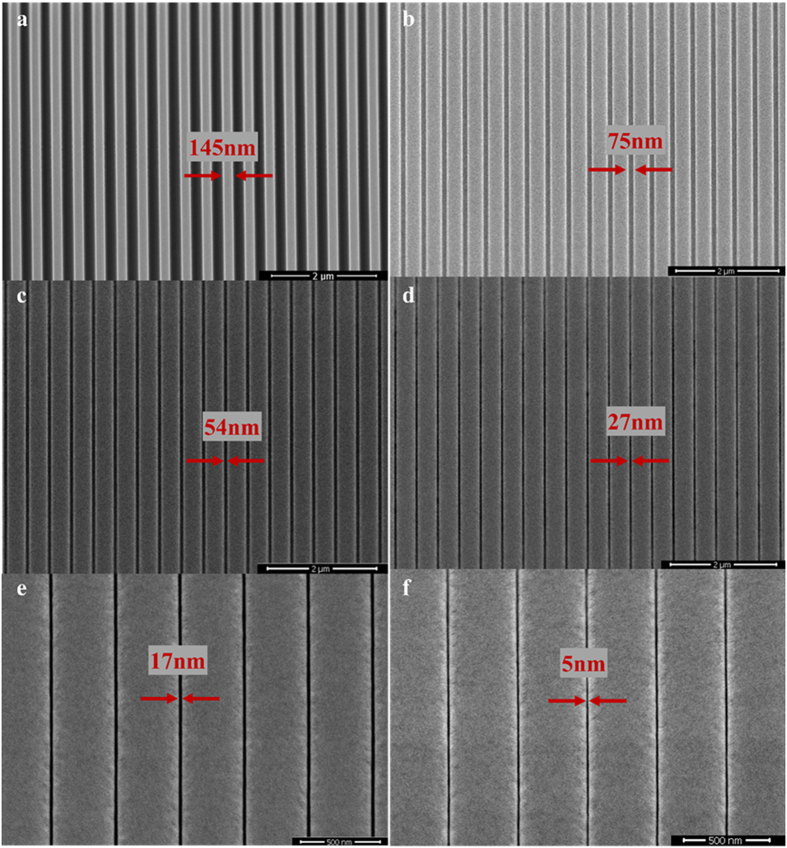
SEM images of the shrinking line array during the ion irradiation. The initial pattern with a gap width of 145 nm before the ion irradiation is shown in (**a**). After merely ~4 minutes of ion exposure, the gap linewidth shrunk to 75 nm (**b**). The images from (**c**–**f**) were taken chronologically after 6, 8.5, 9, and 10 minutes of the ion irradiation, respectively. The result clearly demonstrates the linewidth of the gap shrunk dramatically from 145 nm to 5 nm using the SDML method.

To observe the self-shrinking process more clearly, a series of SEM images were taken during the ion irradiation and are shown in Fig. 3. It is clearly observed that the gap size reduces dramatically from 145 nm to 5 nm with the increase of exposure time. The self-shrinking process shown in Fig. 3 also reveals that the pattern linewidth can be manipulated arbitrarily over a wide range from hundreds to a few nanometers, depending on the ion exposure time. In the supplementary information, Fig. S10 shows the Al_2_O_3_ gap width as a function of the exposure ion dose, which were obtained from the SEM images taken after every dose increment of 55.86 pC/μm^2^ chronologically (shown in Figs S11~S13). It can be clearly seen from these SEM images that the line array is very uniform and nearly free of any defects across a wide horizontal field width (HFW). Figure S10 reveals a linear relation between the gap width reduction and the ion irradiation dose, with a wide range of the gap width from ~120 nm to ~5 nm. The result demonstrates that SDM is a highly controllable method for the precise fabrication of uniform nanopatterns.

**Figure 4:**
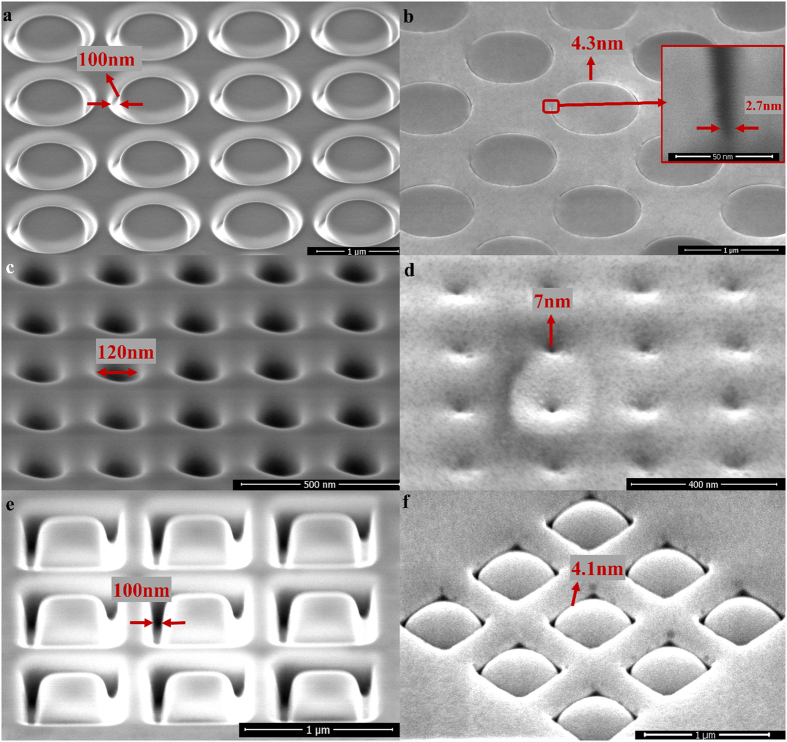
Multiple shapes fabricated by SDM. (**a**) A circle ring array with ~100 nm gap width as the initial pattern prepared by the focused Ga ion beam. (**b**) The gap width of the circle ring was reduced to 4.3 nm after the ion irradiation. The inset shows the cross section of the circle ring, revealing a bottom linewidth down to 2.7 nm. (**c**) A nanohole array with a diameter of ~120 nm as the initial pattern prepared by the focused Ga ion beam. (**d**) The diameter of the nanohole array shrunk to 7 nm after the ion irradiation. (**e**) A square ring array with ~100 nm gap width as the initial pattern prepared by the focused Ga ion beam. (**f**) The gap width of the square ring was reduced to 4.1 nm after the ion irradiation.

Nanopatterns with a variety of different shapes are also feasible to be fabricated by SDM. In Fig. 4a, a circle ring array with a ~100 nm gap width as the initial pattern was fabricated by focused Ga ion beam. A gap width down to 4.3 nm was achieved by SDM as depicted in Fig. 4b. The inset in Fig. 4b is the cross section of the nanogap of the circle ring, revealing that the bottom width of the nanogap is only 2.7 nm. Apart from the circle ring array, initial patterns including a nanohole array with a diameter of ~120 nm and a square ring array with a gap width of ~100 nm were also prepared as shown in Fig. 4c,e, respectively. Figure 4d,f show the diameter of the nanoholes and the gap width of the square ring dramatically shrunk to 7 nm and 4.1 nm, respectively, after the ion irradiation. The result clearly demonstrates that the SDM method is capable of creating nanopatterns with different shapes of a critical dimension down to sub-10 nm scale. It should be noted that the surface of Al_2_O_3_ inside the square become spherical (in Fig. 4e) after the ion irradiation of the square ring patterns.

The most attractive feature of SDM is the self-shrinking process of the hard dielectric mask. There might be a number of mechanisms behind this phenomenon. One of the possible explanation is the re-deposition effect during the ion beam irradiation. That is, the hard dielectric materials are sputtered from the base of the feature onto the sidewalls during the ion irradiation. It has been reported that the holes of the anodic aluminum oxide film were closed after the Ga ion beam irradiation, which was mainly ascribed to the re-deposition of the sidewall alumina to the top capping layer^37^. However, the re-deposition effect cannot explain the whole phenomenon we have observed. For example, Fig. 2b,c clearly shows that the Al_2_O_3_ mask re-shapes from the wide V-shaped geometry to the straight nanogap with a high aspect ratio after the ion irradiation. It is not reasonable that re-deposition would form such a straight gap rather than a V-shaped geometry^33^,^38^,^39^. Furthermore, the spherical Al_2_O_3_ surface as shown in Fig. 4e is unlikely to be solely caused by the re-deposition effect. As a result, re-deposition is not the only mechanism involved in the self-shrinking mask, and some other mechanisms should take place simultaneously. Another likelihood is the ion bombardment induced viscous flow and surface diffusion of the hard dielectric mask, which has been widely discussed in the previous studies^37^,^40^,^41^,^42^,^43^. Please be noticed that the nanopattern as shown in Figs 2 and 3 is quite uniform and straight, which cannot be solely explained by either the re-deposition or the ion-induced viscous flow and surface diffusion. The charging effect might be the possible reason for this high quality nanopattern. When the sample is exposed to the ions, the non-conductive dielectric mask will be charged by positive ions^44^. The charging effect might keep the sputtered or flowing dielectric mask from being severely distorted due to the repulsive force between the positively charged mask. As a result, the line array could stay straight and uniform as shown in in Figs 2 and 3. The TEM image and energy dispersive spectroscopy (EDS) mapping of the nanogap cross section have been provided in the supplementary information. The EDS mapping indicates that the edge of the Al2O3 mask was highly doped by Ga, and so it can be deduced that the Al2O3 mask was charged during the ion irradiation. The TEM image shows that the Ga-doped Al2O3 was filled into the gaps, which might be attributed to the re-deposition or ion-induced viscous flow/surface diffusion. To sum up, the self-shrinking process may be a comprehensive manifestation of the above-mentioned mechanisms. It is noteworthy that the dielectric mask should be hard enough in order to prevent it from being milled out completely. If this happens, self-shrinking process will not occur. Therefore, Al2O3 was chosen as the mask in this study because of its extremely hard-wearing nature to ion milling.

**Figure 5:**
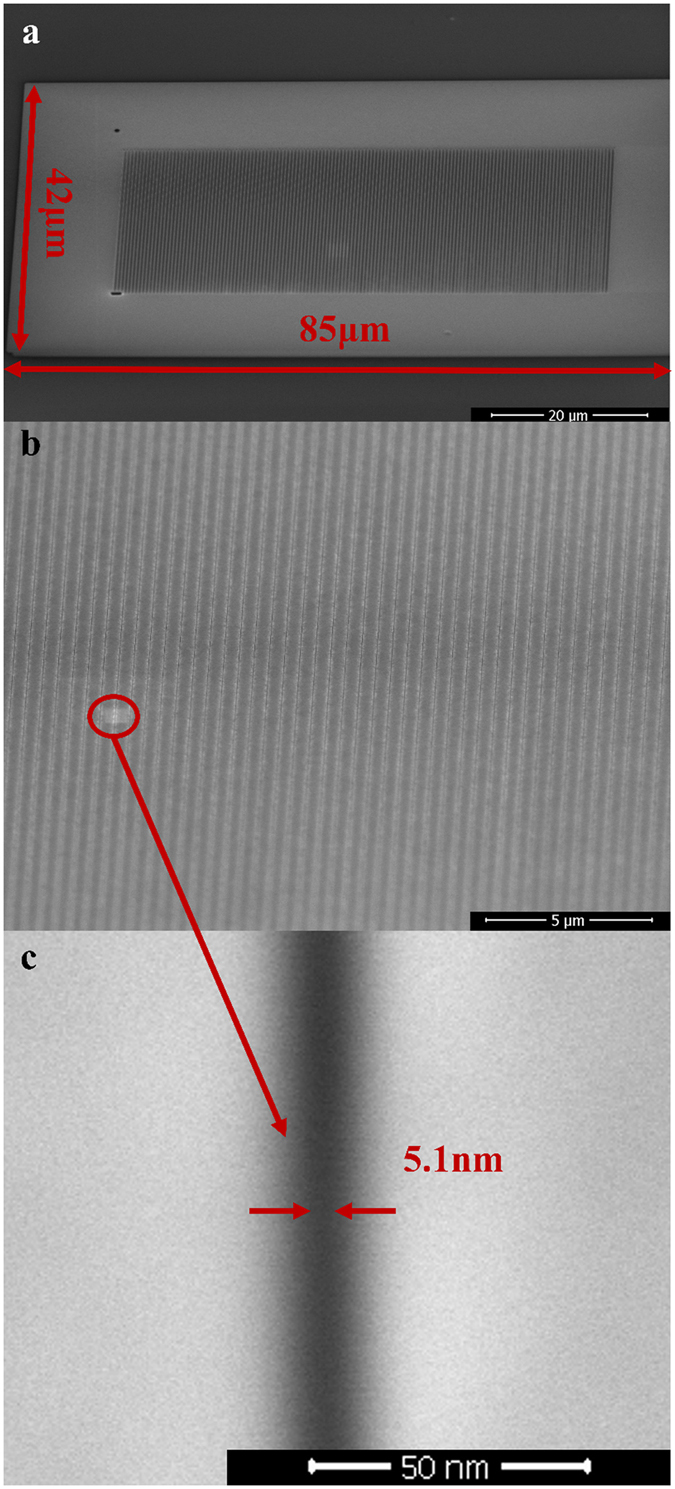
SEM images of a large area of uniform ~5 nm patterns fabricated by the SDM method. (**a**) A large exposure area of ~3570 μm^2^ with the ion irradiation. (**b**) A magnified image from (**a**), revealing a uniform line array over a large area. (**c**) A magnified image from (**b**), revealing a narrow gap of only 5.1 nm in width.

Another attractive point of SDM is its capability to fabricate a myriad of nanopatterns simultaneously as long as the initial patterns are within the exposure area of the ion source. In Fig. 5, its ability to fabricate a large area of a uniform sub-10 nm line array is shown. A large exposure area of 3570 μm^2^ irradiated by the Ga ions is illustrated in Fig. 5a. In fact, even a larger area can be achieved by SDM as long as the initial pattern is within the exposure region of the ion source. Figure 5b depicts the uniformly fabricated sub-10 nm line array after the ion irradiation, and a small linewidth of only 5.1 nm is clearly manifested in Fig. 5c.

Some particular methods had been proposed to define sub-10 nm patterns by focused ion beam, such as delineating patterns on a sample with a thick sacrificial metal layer using the lowest Ga ion beam current^45^, or utilizing helium or neon as the ion beam source^10^,^11^. However, these techniques are extremely time-consuming with a very low throughput to fabricate nanostructures within a large area, because of the low beam current (1.1pA) and the inefficient milling rate of helium and neon. For example, to fabricate a line array with a large area of 3570 μm^2^ (the same as that shown in Fig. 5a) with sequential line-by-line writing by focused Ga ion beam, it will take more than 610 hours using the lowest 1.1 pA beam current. Nevertheless, the minimum gap width is ~20 nm even using this lowest beam current. However, it takes only ~30 minutes (less than 20 minutes for the initial patterns and about 10 minutes for the ion irradiation) to fabricate a sub-10 nm (~5.1 nm) line array with a large area of 3570 μm^2^ using the SDM technique (Fig. 5). This result indicates that SDM is over 1000 times more rapid than the focused ion beam lithography and the feature size down to a few nanometer can be achieved at the same time.

In conclusion, we have proposed a novel concept to fabricate sub-3 nm patterns. Based on the self-shrinking mask instead of the focused beam approach, the SDM method can fabricate nanopatterns with the feature size of only a few nanometer, which is difficult to be fulfilled with the conventional lithography. We believe that the SDM technique opens a new door and impinges a great impact on the nanofabrication of sub-5 nm patterns in a variety of applications.

## Methods

### Preparation of the Al_2_O_3_ dielectric mask

The Al_2_O_3_ layer of a thickness of ~360 nm was deposited by atomic layer deposition at 200** °C** (Savannah S100, Cambridge nanotech), using trimethylaluminum [TMA, Al(CH_3_)_3_], and H_2_O vapor as the precursors.

### Initial patterns on the Al_2_O_3_ dielectric mask

A line array or other initial patterns on the Al_2_O_3_ dielectric mask can be prepared by conventional lithography techniques. In this study, we delineated the initial patterns on the Al_2_O_3_ mask by the focused Ga ion beam (FEI Helios Nanolab 600i dual beam system) with an accelerating voltage of 30 kV, a beam current of 2.5 nA, 1 μs dwell time, and a dose of 2.4 nC/μm^2^ for each gap.

### Ion irradiation

The samples with initial patterns on the Al_2_O_3_ mask was irradiated by Ga ions at 30 keV and a beam current of 2.5 nA (FEI Helios Nanolab 600i dual beam system). The samples were exposed to Ga ions with a field of view of 3570 μm^2^ for about 10 minutes to get sub-3 nm nanopatterns on the Al_2_O_3_ mask. *In situ* observation was carried out by the SEM equipped in the dual beam system.

### Wet etching of the Al_2_O_3_ mask

A_l2_O_3_ was etched away in dilute HF solution. Then the sample was observed by the SEM equipped in FEI Helios 600i dual beam system.

## Additional Information

**How to cite this article**: Yang, P.-S. *et al.* Novel Self-shrinking Mask for Sub-3 nm Pattern Fabrication. *Sci. Rep.* **6**, 29625; doi: 10.1038/srep29625 (2016).

## Supplementary Material

Supplementary Information

## References

[b1] Scholder O. *et al.* Helium focused ion beam fabricated plasmonic antennas with sub-5 nm gaps. Nanotechnology 24, 395301 (2013). 24013454 10.1088/0957-4484/24/39/395301

[b2] Wang Y. *et al.* Ultrafast nonlinear control of progressively loaded, single plasmonic nanoantennas fabricated using helium ion milling. Nano letters 13, 5647–5653 (2013). 24127754 10.1021/nl403316z

[b3] Tang X., Francis L., Dutu C. A., Reckinger N. & Raskin J.-P. InNanotechnology (IEEE-NANO), 2013 13th IEEE Conference on 570–573 (IEEE, 2013).

[b4] Wang X. *et al.* Room-temperature all-semiconducting sub-10-nm graphene nanoribbon field-effect transistors. Physical review letters 100, 206803 (2008). 18518566 10.1103/PhysRevLett.100.206803

[b5] Siegfried T., Ekinci Y., Solak H., Martin O. J. & Sigg H. Fabrication of sub-10 nm gap arrays over large areas for plasmonic sensors. Applied Physics Letters 99, 263302 (2011).

[b6] Zhu W., Banaee M. G., Wang D., Chu Y. & Crozier K. B. Lithographically fabricated optical antennas with gaps well below 10 nm. Small 7, 1761–1766 (2011). 21591254 10.1002/smll.201100371

[b7] Duan H., Hu H., Kumar K., Shen Z. & Yang J. K. Direct and reliable patterning of plasmonic nanostructures with sub-10-nm gaps. ACS nano 5, 7593–7600 (2011). 21846105 10.1021/nn2025868

[b8] Bisio F. *et al.* Pushing the high-energy limit of plasmonics. ACS nano 8, 9239–9247 (2014). 25181497 10.1021/nn503035b

[b9] Boden S., Moktadir Z., Bagnall D., Mizuta H. & Rutt H. Focused helium ion beam milling and deposition. Microelectronic Engineering 88, 2452–2455 (2011).

[b10] Winston D. *et al.* Neon Ion Beam Lithography (NIBL). Nano letters 11, 4343–4347 (2011). 21899279 10.1021/nl202447n

[b11] Sidorkin V. *et al.* Sub-10-nm nanolithography with a scanning helium beam. Journal of Vacuum Science & Technology B 27, L18–L20 (2009).

[b12] Hu W., Sarveswaran K., Lieberman M. & Bernstein G. H. Sub-10 nm electron beam lithography using cold development of poly(methylmethacrylate). Journal of Vacuum Science & Technology B: Microelectronics and Nanometer Structures 22, 1711 (2004).

[b13] Vieu C. *et al.* Electron beam lithography: resolution limits and applications. Applied Surface Science 164, 111–117 (2000).

[b14] Broers A., Hoole A. & Ryan J. Electron beam lithography—Resolution limits. Microelectronic Engineering 32, 131–142 (1996).

[b15] Manfrinato V. R. *et al.* Resolution limits of electron-beam lithography toward the atomic scale. Nano letters 13, 1555–1558 (2013). 23488936 10.1021/nl304715p

[b16] Van Dorp W. F., van Someren B., Hagen C. W., Kruit P. & Crozier P. A. Approaching the resolution limit of nanometer-scale electron beam-induced deposition. Nano letters 5, 1303–1307 (2005). 16178228 10.1021/nl050522i

[b17] Garcia R., Knoll A. W. & Riedo E. Advanced scanning probe lithography. Nat Nano 9, 577–587 (2014). 10.1038/nnano.2014.157 25091447

[b18] Austin M. D. *et al.* Fabrication of 5 nm linewidth and 14 nm pitch features by nanoimprint lithography. Applied Physics Letters 84, 5299–5301 (2004).

[b19] Stephen Y. Chou P. R. K., Wei Zhang, Lingjie Guo & Zhuang Lei Sub-10 nm imprint lithography and applications. Journal of Vacuum Science & Technology B 15 (1997).

[b20] Wagner C. & Harned N. EUV lithography: Lithography gets extreme. Nature Photonics 4, 24–26 (2010).

[b21] Bakshi V. EUV lithography, Vol. 178. (Spie Press Bellingham, 2009).

[b22] Bjorkholm J. E. EUV lithography—the successor to optical lithography? Intel Technology Journal 3, 98 (1998).

[b23] Pease R. F. Maskless lithography. Microelectronic Engineering 78–79, 381–392 (2005).

[b24] Reyntjens S. & Puers R. A review of focused ion beam applications in microsystem technology. Journal of Micromechanics and Microengineering 11, 287 (2001).

[b25] Tseng A. A. Recent developments in micromilling using focused ion beam technology. Journal of Micromechanics and Microengineering 14, R15 (2004).

[b26] Kim C.-S., Ahn S.-H. & Jang D.-Y. Review: Developments in micro/nanoscale fabrication by focused ion beams. Vacuum 86, 1014–1035 (2012).

[b27] Utke I., Moshkalev S. & Russell P. Nanofabrication using focused ion and electron beams: principles and applications. (Oxford University Press, 2012).

[b28] Bakshi V. EUV source technology. EUV lithography. SPIE: Bellingham, WA, (2009).

[b29] Hill S. *et al.* In SPIE Advanced Lithography 692117-692117-692111 (International Society for Optics and Photonics, 2008).

[b30] Pan D. Z., Liebmann L., Bei Y., Xiaoqing X. & Yibo L. In 2015 52nd ACM/EDAC/IEEE Design Automation Conference (DAC) 1–6 (2015).

[b31] Moon H. S. *et al.* Atomic layer deposition assisted pattern multiplication of block copolymer lithography for 5 nm scale nanopatterning. Advanced Functional Materials 24, 4343–4348 (2014).

[b32] Philipp G., Weimann T., Hinze P., Burghard M. & Weis J. Shadow evaporation method for fabrication of sub 10 nm gaps between metal electrodes. Microelectronic engineering 46, 157–160 (1999).

[b33] Giannuzzi L. A. & Stevie F. A. Introduction to focused ion beams: instrumentation, theory, techniques and practice. (Springer Science & Business Media, 2005).

[b34] Wang Y. M. *et al.* High aspect ratio 10-nm-scale nanoaperture arrays with template-guided metal dewetting. Sci. Rep. 5, 10.1038/srep09654 (2015). PMC4392361 25858792

[b35] Melngailis J. Focused ion beam technology and applications. Journal of Vacuum Science & Technology B 5, 469–495 (1987).

[b36] Hopman W. C. *et al.* Focused ion beam scan routine, dwell time and dose optimizations for submicrometre period planar photonic crystal components and stamps in silicon. Nanotechnology 18, 195305 (2007).

[b37] Liu N. W. *et al.* Fabrication of Anodic‐Alumina Films with Custom‐Designed Arrays of Nanochannels. Advanced Materials 17, 222–225 (2005).

[b38] Lugstein A., Basnar B., Smoliner J. & Bertagnolli E. FIB processing of silicon in the nanoscale regime. Applied Physics A 76, 545–548 (2003).

[b39] Kubena R., Seliger R. & Stevens E. High resolution sputtering using a focused ion beam. Thin Solid Films 92, 165–169 (1982).

[b40] Makeev M. A. & Barabási A.-L. Ion-induced effective surface diffusion in ion sputtering. Applied physics letters 71, 2800–2802 (1997).

[b41] Facsko S. *et al.* Formation of ordered nanoscale semiconductor dots by ion sputtering. Science 285, 1551–1553 (1999). 10477516 10.1126/science.285.5433.1551

[b42] Mayer T., Chason E. & Howard A. Roughening instability and ion‐induced viscous relaxation of SiO2 surfaces. Journal of applied physics 76, 1633–1643 (1994).

[b43] Vauth S. & Mayr S. Relevance of surface viscous flow, surface diffusion, and ballistic effects in keV ion smoothing of amorphous surfaces. Physical Review B 75, 224107 (2007).

[b44] Kim K. H., Akase Z., Suzuki T. & Shindo D. Charging effects on SEM/SIM contrast of metal/insulator system in various metallic coating conditions. Materials transactions 51, 1080–1083 (2010).

[b45] Menard L. D. & Ramsey J. M. Fabrication of sub-5 nm nanochannels in insulating substrates using focused ion beam milling. Nano letters 11, 512–517 (2011). 21171628 10.1021/nl103369g PMC3125600

