# Supplementary material for: Novel Self-shrinking Mask for Sub-3 nm Pattern Fabrication
Source: Sci Rep. 2016 Jul 12;6:29625. doi: 10.1038/srep29625 (PMC4940743; doi:10.1038/srep29625)
Supplement: Supplementary Information [file srep29625-s1.pdf]

Novel self-shrinking mask for sub-3nm pattern fabrication

Supplementary information

Po-Shuan.Yang[1], Po-Hsien. Cheng [1], C. Robert. Kao[1], and Miin-Jang. Chen[1]*

[1] Department of Materials Science and Engineering National Taiwan University

1, Roosevelt Road, Sec. 4, Taipei, Taiwan 106, ROC

*Corresponding Author, E-mail: mjchen@ntu.edu.tw Phone: +886-2-3366-5301 Fax: +886-2-2363-4562

1. Junctionless Si transistors on SOI (silicon on insulator) substrate with a sub-10nm gate defined by the SDM method

The SEM image of the transistor structure is shown in Fig. S1. A *p*-type SOI wafer with a top Si layer of ~20 nm was used as the substrate. The source, channel, and drain regions were defined by electron beam lithography. Pt was deposited on the source and drain as the metal contact. A ZrO_2 layer was deposited by atomic layer deposition (ALD) on the channel as the high-*K* gate oxide, and then an Al_2O_3 layer was prepared upon the ZrO_2 as the dielectric mask for SDM. Then we used the SDM technique to create a sub-10nm gap with a feature size about 9.7 nm, as shown in the inset of Fig. S1. Afterwards a TiN metal gate was deposited by ALD into the nanogap, and thus a sub-10nm gate was achieved. The schematic of the junctionless Si transistor with a sub-10nm gate length defined by SDM is illustrated in Fig. S2. Figure S3 shows the drain current versus gate voltage (I_{DS}-V_{GS}) curves of the transistor, revealing a current on/off ratio of ~3×10^4. The result manifestly indicates that the SDM method is practically applicable and has been successfully implemented to define the critical dimension of the nanoscale transistors, which opens a promising opportunity and possibility for this technique in a variety of applications.

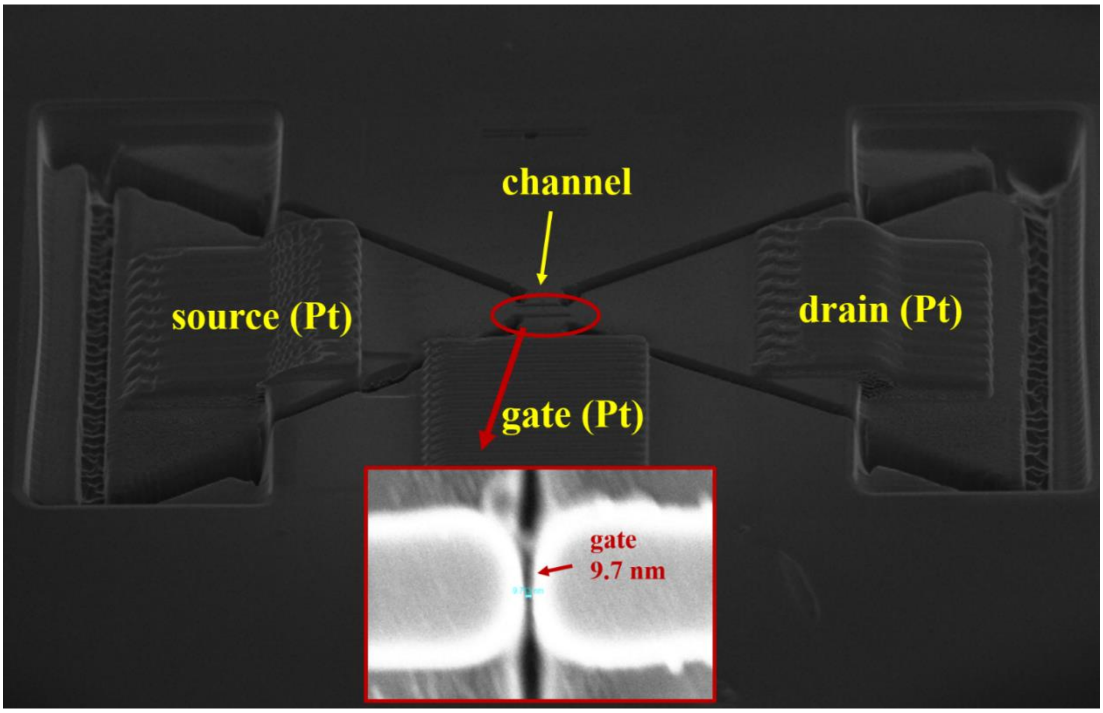

Figure S1. The SEM image of the junctionless Si transistors on SOI, with a sub-10nm gate created by the SDM technique.

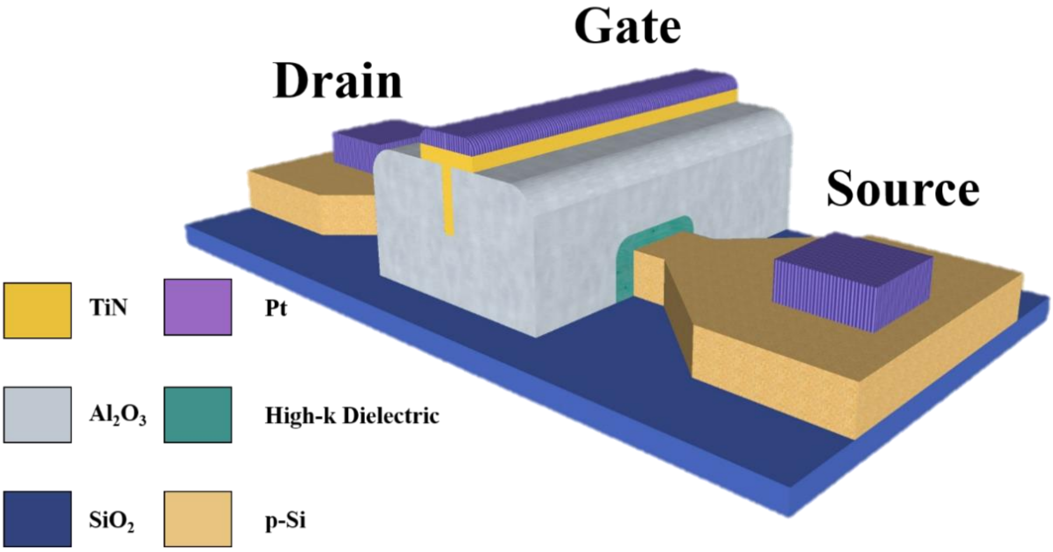

Figure S2. Schematic of the nanoscale junctionless Si transistor.

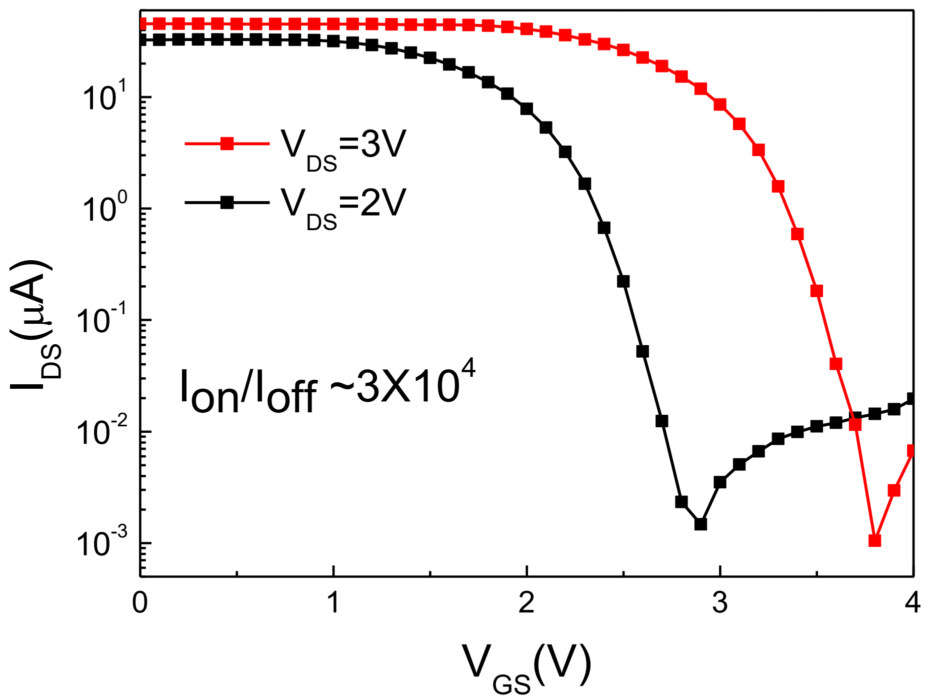

Figure S3. The drain current versus gate voltage (I_{DS}-V_{GS}) curves of the junctionless Si transistor on SOI. A positive gate voltage was applied to deplete the carriers in the *p*-type top Si layer on SOI to turn off the transistor.

2. Pitch reduction by multiple patterning

In the SDM technique, the pitch denity is the same as that of the initial pattern when the patterns shrink during the ion irradiation. This could be a major problem for SDM to be practically implemented in the future high-density patterning. However, the pitch can be reduced by multiple patterning as shown schematically in Fig. S4. An additional hard dielectric mask layer is deposited on the patterns which have been fabricated by SDM. Afterwards, an extra initial pattern is created on the hard mask between the existing SDM patterns, and then another SDM process is applied. In the end, the hard dielectric mask is removed and thus the patterns with smaller pitch can be achieved. The multiple patterning can be repeated until the desired pitch is fulfilled.

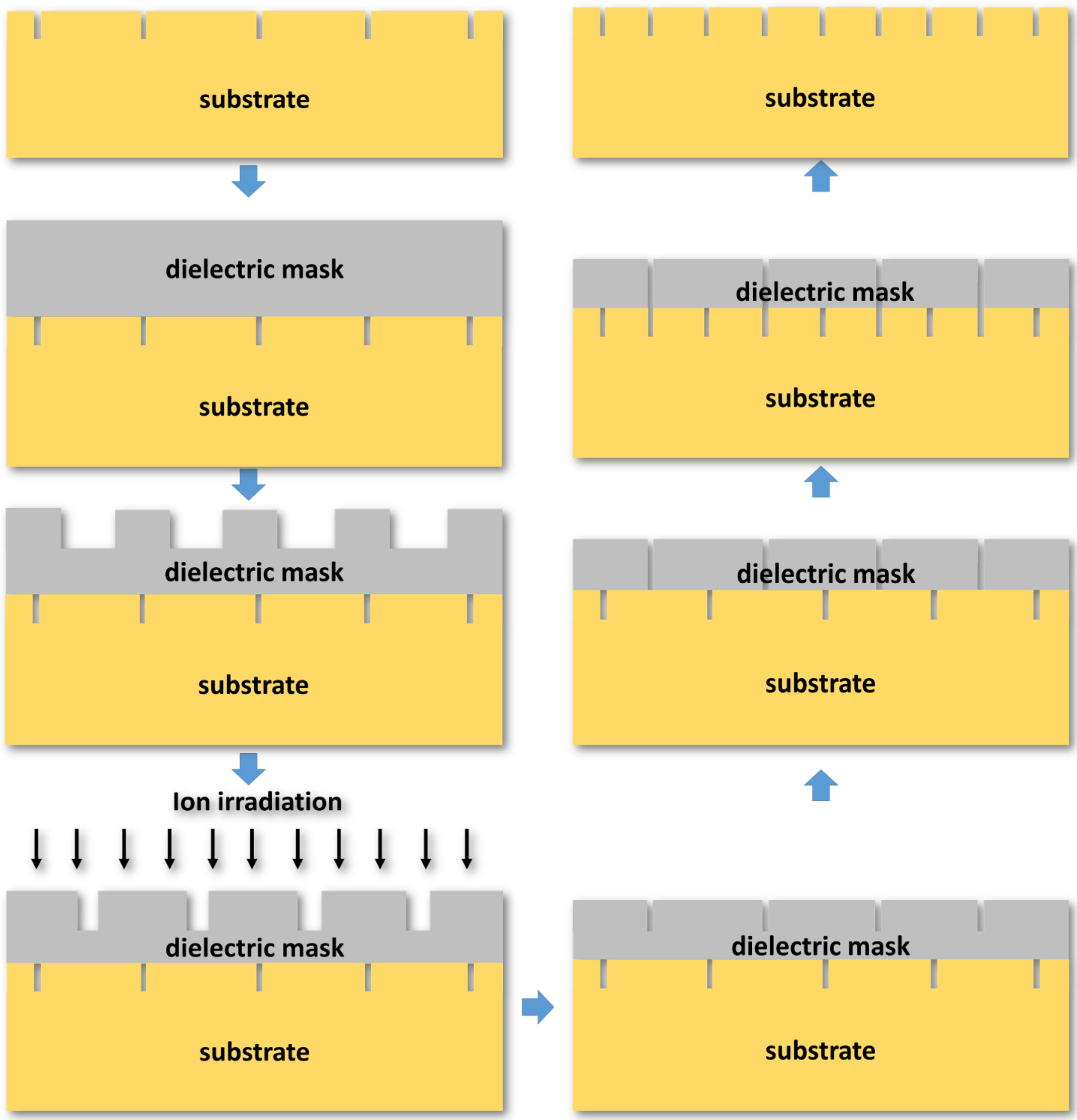

Figure S4. Schematic of the double patterning for SDML. Smaller pitch can be fulfilled by multiple patterning.

3.The line edge roughness of the SDM line patterns

The line-edge roughness has been measured by the software Image J with a plugin function “Analyze_Stripes”. The SEM image of the Al2O3 nanogap array for the line-edge roughness analysis was provided in Fig. R8, in which the linewidth of the gaps is ~ 5 nm and the area of the analyzed region is about 4μm×2.2μm. The analysis indicates that root-mean-square(RMS) edge roughness is onlly 1.4nm.

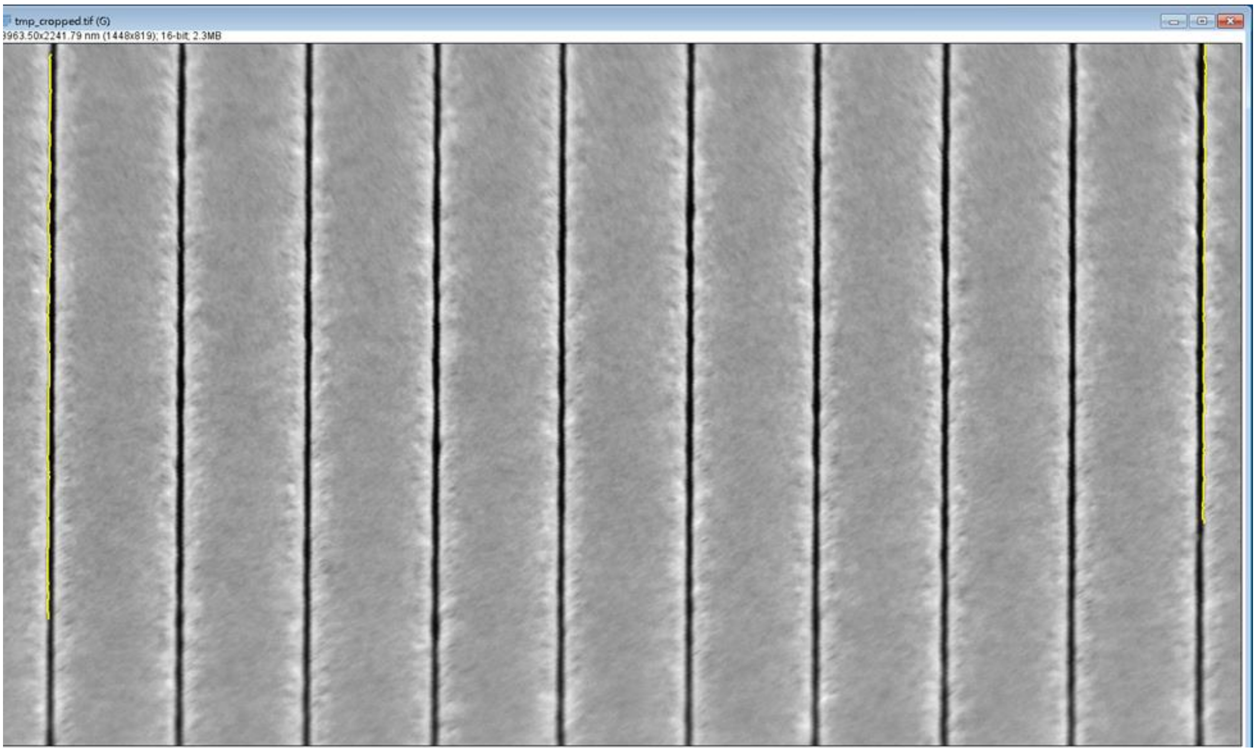

RMS edge roughness (Rq)= 1.402E0 nm

Figure S5. Calculation of the edge roughness of the SDM line array

4.The TEM pictures and analysis of the SDM process

We made the TEM sample of Fig. S5 by the Helios 600i dual beam system. The cross-section of the sample has been shown in Fig. S6. The morphology is a little bit different from the one in Fig. 2c. The reason is that we deposited the alumina mask thinner than the one in the article to research for the mechanism of the SDM process. As we can see in Fig. S6, the Ga ion penetrated through the alumina mask and etched into the Si substrate before the mask shrunk down to sub-10nm. However, the shrinking alumina mask still protects the psttern line on the Si from broadening. As a result, sub-5nm gaps on Si can still be fabricated as shown in Fig. S7. In Fig. S8, the STEM image of the

SDM cross-section is shown. In the image, the curtain effect is clearly observed and consequently the gaps seem deeper than they should be. There is a C protective layer on the alumina mask and the gaps on the surface were filled during the deposition of the protective layer so that originally there is a straight gap from the Si bottom to the alumina surface. We deliberately made the alumina mask thinner (200nm)to see the interaction of the mask and the Si more clearly. It seems that the sputtered alumina re-deposited into the Si gap and protected it from being larger. Furthermore, in Fig. S9, we can see the EDAX mapping of the SDM cross-section. The alumina mask was highly doped by the Ga ion whether it is on the top of the Si or in the Si gap. As a result, it is highly possible that the mask was charged during the ion irradiation and made the alumina gap straighter than it should be.

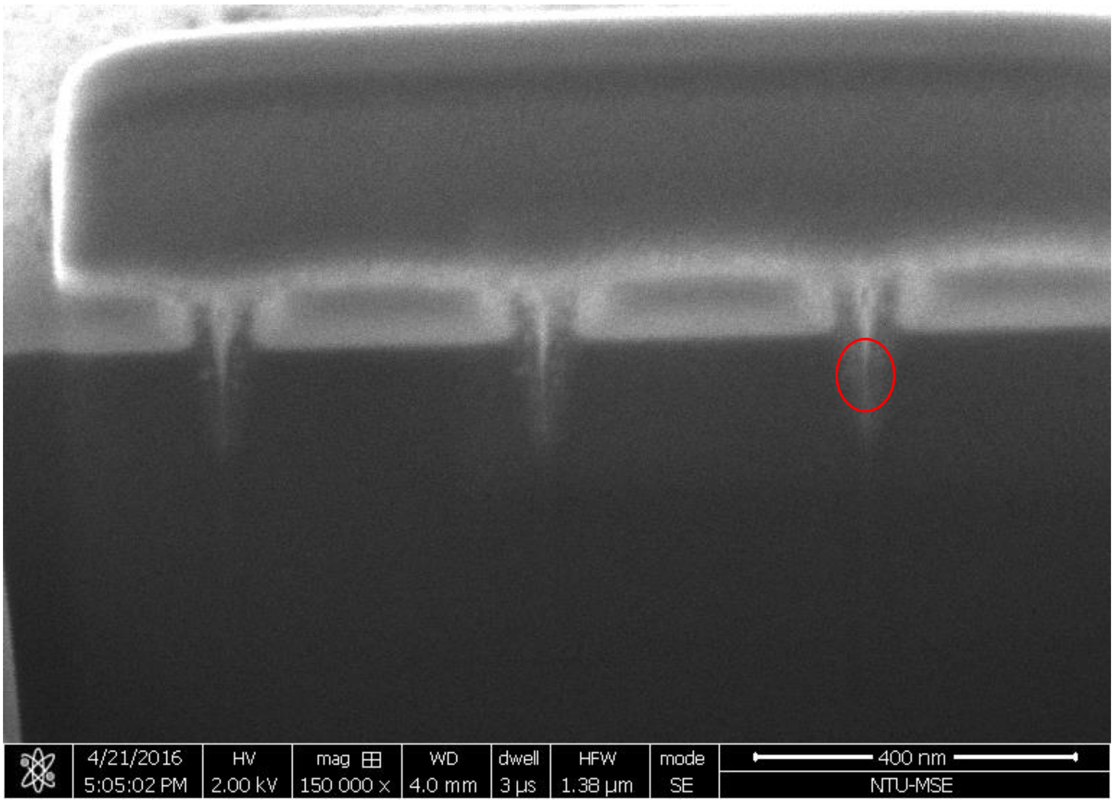

Figure S6.SEM image of the cross-section of the SDM

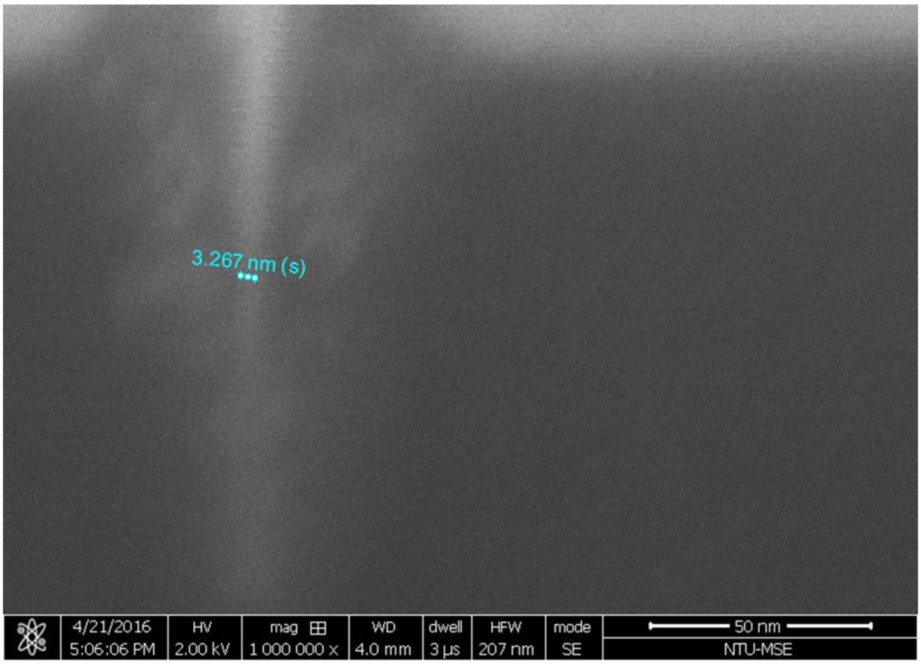

Figure S7. The magnification image of the selected area in Fig. S6

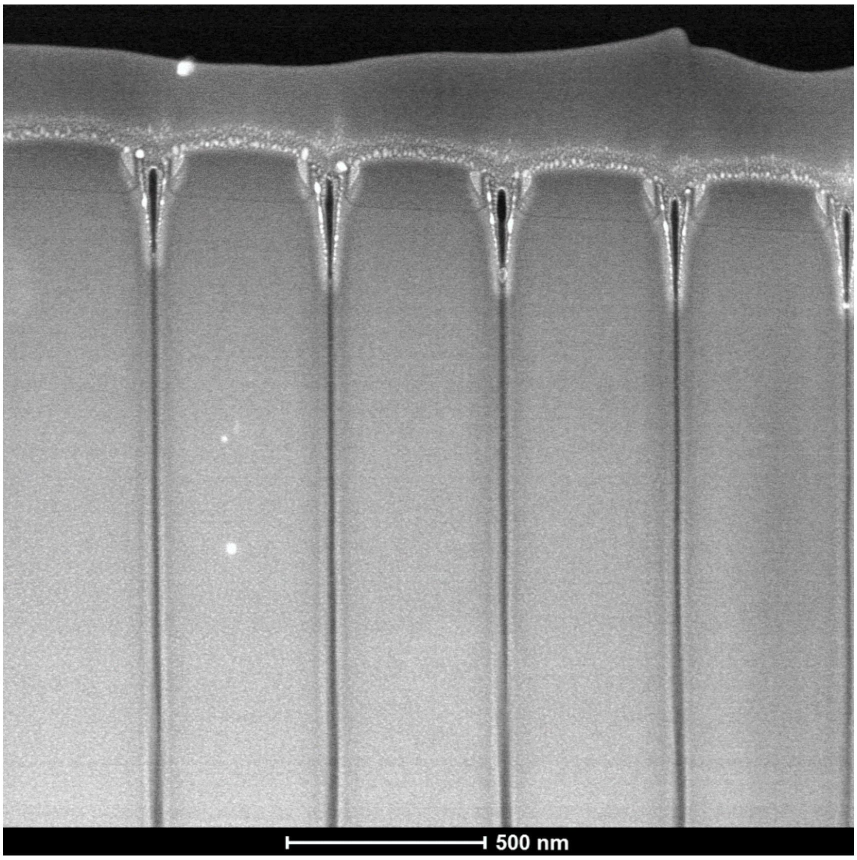

Figure S8. STEM image of the SDM cross-section. The depth of the gap is not as deep as what the Fig. S6 shown. The reason why the gaps seem so deep in Fig. S8 is that when the TEM sample was fabricated, the milling angle of the Ga ion was changing frequently. As a result,the Ga ions were not always perpendiculer to the sample and the

Ga ions may scratch along the gap to form the fake gap image.This effect is also called the “curtain effect”.

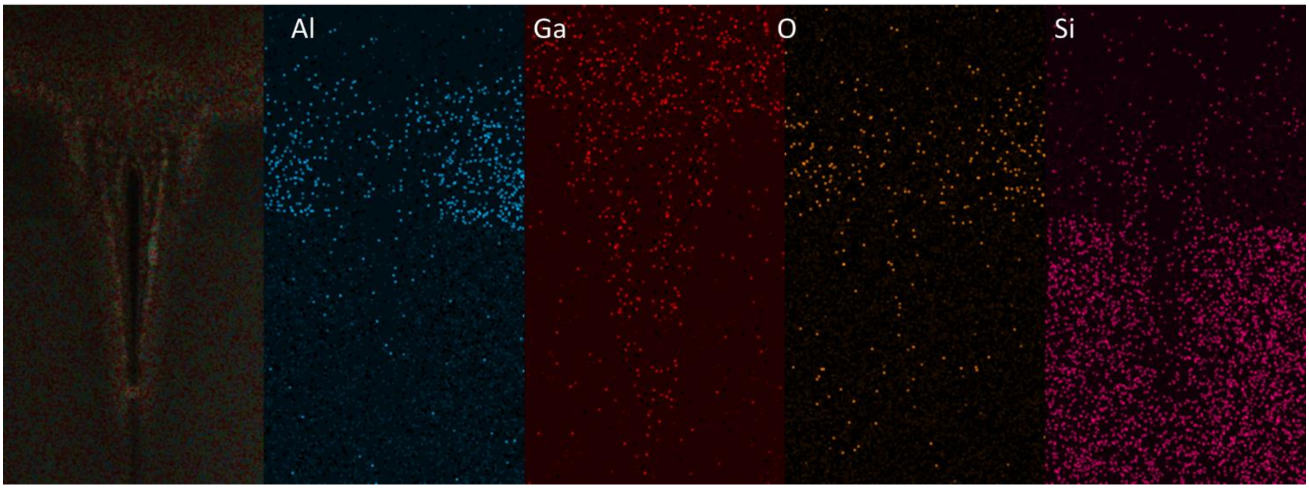

Figure. S9 EDX mapping of the shrunk gap

5.Uniformity and controllability of the SDM proscess

We repeated the experiment and took the SEM photos after certain amount of the dose chronologically.We took the picture after every dose of 55.86 pC/um^2 and draw a relation between the ion irradiation dose and the gap width as shown in Fig. S10. We stopped the ion dose at about 530.67 pC/um^2 because we reach our target linewidth at about 5 nm. In Fig. S11-S13, the SEM images of the SDM gap width after certain amount of the ion dose were shown. In these photos, we can clearly see that with a field of view of nearly 20um,the line array is very uniform and nearly without any defects after each amount of the ion dose irradiation. As a result, we think that SDM is a highly controllable and uniform method for nanopatterns fabrication.

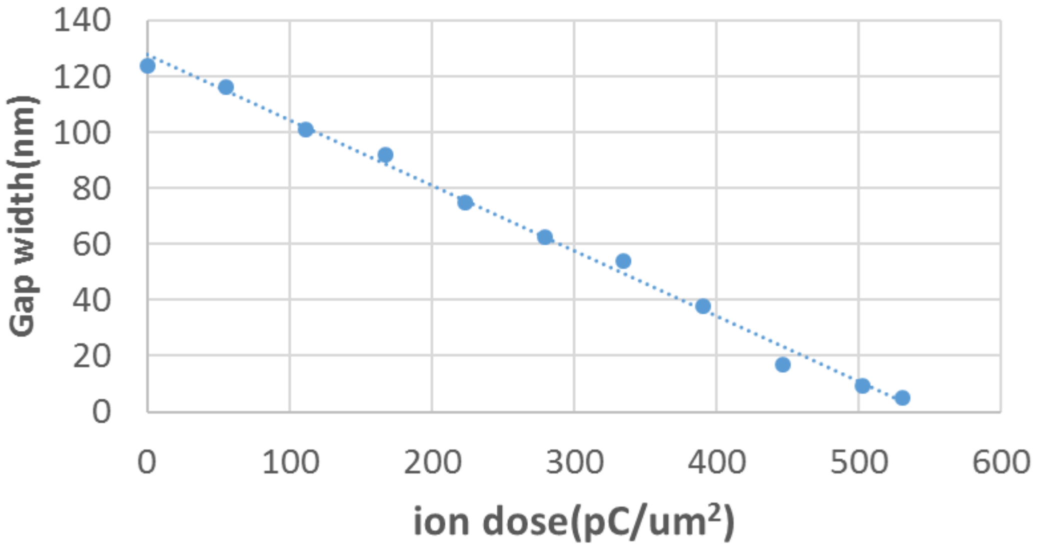

Figure S10. The relation between the change of the gap width and the ion irrdiation dose.

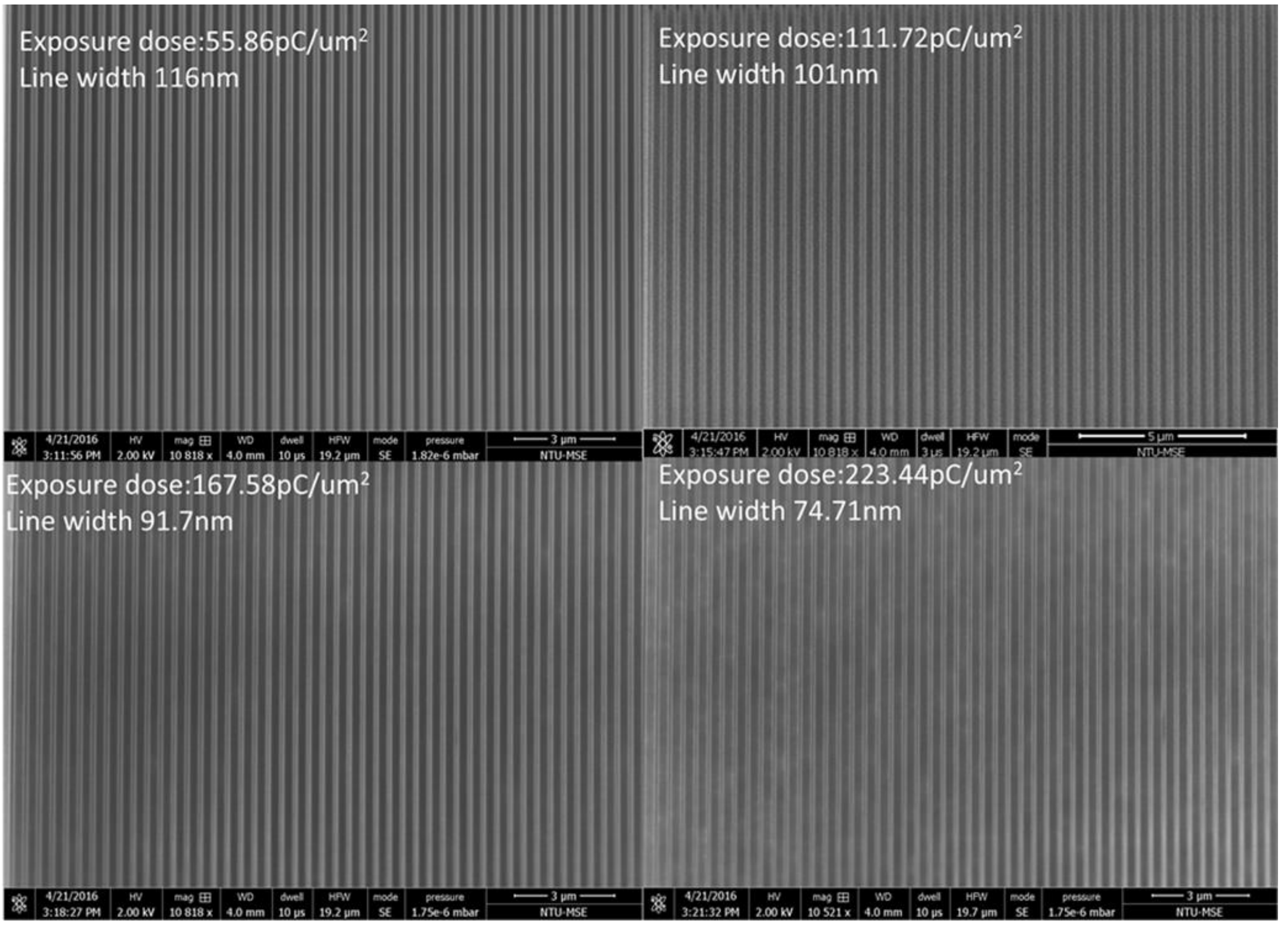

Figure S11. SEM photos of the exposure dose versus the linewidth

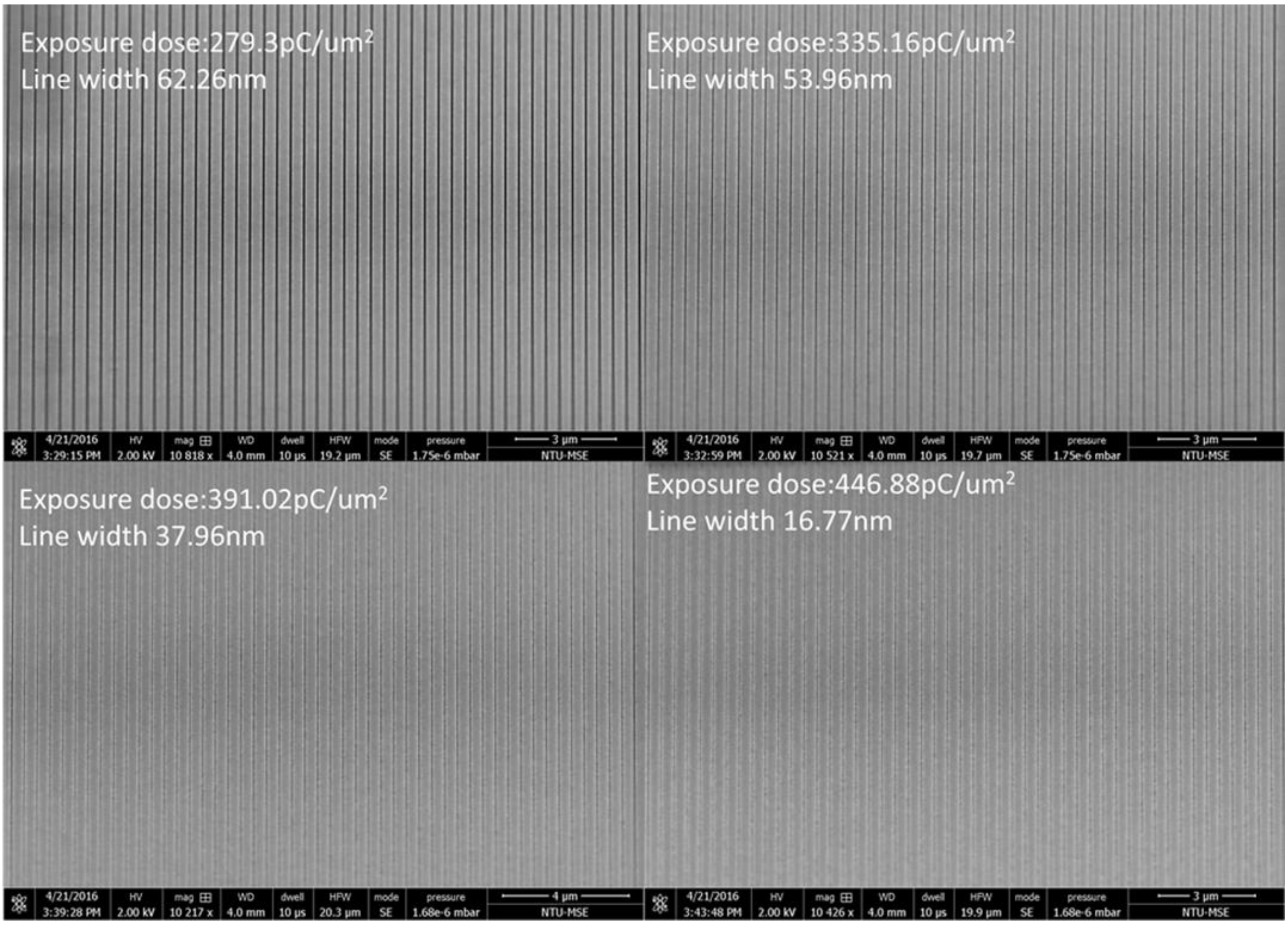

Figure S12. SEM photos of the exposure dose versus the linewidth

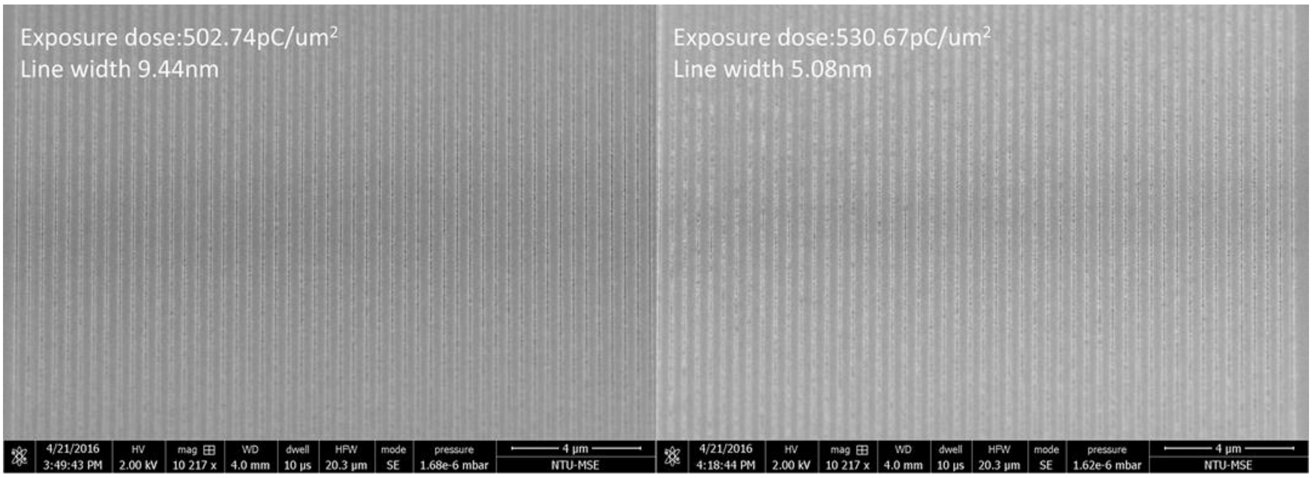

Figure S13. SEM photos of the exposure dose versus the linewidth
